# Cost–benefit ratio of modern medical education using micro-costing: a model calculation using the example of an innovative breast brachytherapy workshop

**DOI:** 10.1007/s00066-024-02218-6

**Published:** 2024-02-28

**Authors:** Stefan Knippen, Marciana-Nona Duma, Michael Schwedas, Steffen Schrott, Sonja Drozdz, Irina Mäurer, Guido Hildebrandt, Matthias Mäurer

**Affiliations:** 1 Department of Radiation Oncology, Helios Clinics of Schwerin—University Campus of MSH Medical School Hamburg, Schwerin, Germany; 2 Department for Human Medicine, MSH Medical School Hamburg, Hamburg, Germany; 3 Department of Radiation Oncology, University Medical Center Jena, Jena, Germany; 4 Department for Neurology, University Medical Center Jena, Jena, Germany; 5 Department of Radiation Oncology, University Medical Center Rostock, Rostock, Germany

**Keywords:** Teaching, Cost efficiency, Simulation, Students, Training

## Abstract

**Background and purpose:**

Radiation oncology is an essential component of therapeutic oncology and necessitates well-trained personnel. Multicatheter brachytherapy (MCBT) is one radiotherapeutic option for early-stage breast cancer treatment. However, specialized hands-on training for MCBT is not currently included in the curriculum for residents. A recently developed hands-on brachytherapy workshop has demonstrated promising results in enhancing knowledge and practical skills. Nevertheless, these simulation-based teaching formats necessitate more time and financial resources. Our analyses include computational models for the implementation and delivery of this workshop and can serve as a basis for similar educational initiatives.

**Methods:**

This study aimed to assess the cost-effectiveness of a previously developed and evaluated breast brachytherapy simulation workshop. Using a micro-costing approach, we estimated costs at a detailed level by considering supplies, soft- and hardware, and personnel time for each task. This method also allows for a comprehensive evaluation of the costs associated with implementing new medical techniques. The workshop costs were divided into two categories: development and workshop execution. The cost analysis was conducted on a per-participant basis, and the impact on knowledge improvement was measured using a questionnaire.

**Results:**

The total workshop costs were determined by considering the initial workshop setup expenses including the development and conceptualization of the course with all involved collaborators, as well as the costs incurred for each individual course. The workshop was found to be financially efficient, with a per-participant cost of € 39, considering the industrial sponsorship provided for brachytherapy equipment. In addition, we assessed the workshop’s efficacy by analyzing participant feedback using Likert scale evaluations. The findings indicated a notable enhancement in both theoretical and practical skills among the participants. Moreover, the cost-to-benefit ratio (CBFR) analysis demonstrated a CBFR of € 13.53 for each Likert point increment.

**Conclusion:**

The hands-on brachytherapy workshop proved to be a valuable and approximately cost-effective educational program, leading to a significant enhancement in the knowledge and skills of the participants. Without the support of industrial sponsorship, the costs would have been unattainable.

**Supplementary Information:**

The online version of this article (10.1007/s00066-024-02218-6) contains supplementary material, which is available to authorized users.

## Introduction

Radiation oncology is an important component of therapeutic oncology and requires qualified personnel. As such, it should be properly incorporated into medical education [1, 2]. Accelerated partial breast irradiation (APBI) using interstitial multicatheter brachytherapy (MCBT) is a short and highly effective treatment for breast cancer [3, 4]. APBI can also be delivered with external beam radiation therapy. Even though there are guidelines for target volume delineation [5], volumes may vary among different observers and even among experienced ones [6]. Moreover, since the publication of the Groupe Européen de Curiethérapie and the European Society for Radiotherapy & Oncology (GEC-Estro trial), which has been now updated for 10 years of follow-up data [7], most of the guidelines mention MCBT-APBI as a valid option. MCBT allows for complex 3D dose shaping, resulting in lower doses to surrounding structures and organs at risk compared to standard whole-breast radiation therapy [8]. In fact, it is possible to achieve very low doses to the heart in daily practice [9]. Additionally, MCBT is a short treatment that is typically delivered within 1 week, compared to the 3–5 weeks required for whole-breast irradiation (WBI). Despite being an invasive procedure, MCBT does not lead to a clinically significant decrease in quality of life compared to WBI [10]. However, the hands-on nature of MCBT necessitates procedural training.

Brachytherapy training is recognized as an essential topic during radiation oncology residency. However, only 54% of residents express confidence in developing brachytherapy practice, compared to 97% who are confident in stereotactic techniques. Interestingly, residents feel more confident in gynecologic and prostate brachytherapy than in breast brachytherapy [11]. A recent survey of radiation oncology residents in Europe found that 60% of respondents consider performing brachytherapy independently at the end of residency important. Unfortunately, respondents also reported barriers to achieving independence in brachytherapy due to inadequate didactic and procedural training [12]. Consequently, there is a strong demand for high-quality training in the field of radiation oncology. Simulation training in health profession education is associated with knowledge and skills outcomes [13]. Moreover, for the US, Zaorsky et al. described some knowledge gaps and misconceptions of radiation oncology among medical students and primary care physicians [14]. For Canada, Loewen et al. described that the demand for radiation oncologists is expected to grow more quickly than future expansion in staffing levels [15]. New teaching concepts could arouse interest in future residents [16]. Dedicated hands-on workshops can help to address the significant interest in brachytherapy among residents and the perceived lack of training opportunities [17]. However, it is important to acknowledge that intensive teaching incurs costs [18].

We recently designed a hands-on workshop for medical students and residents to teach them breast cancer brachytherapy. The workshop consisted of a lesson on breast cancer background knowledge and treatment, followed by a 60-minute practical hands-on session using silicone breast models and real brachytherapy equipment. The results of an evaluation questionnaire that tracked participants’ knowledge progression have been published elsewhere. Participants showed significant improvements in their knowledge-based and practical skills in all areas, as assessed by a standardized questionnaire [19]. However, teaching does come with costs. Teaching is a time-consuming process, and in a hospital, any time-dependent process incurs costs. Canadian teaching hospitals have higher wage rates and higher costs due to a larger number of beds [20]. In Germany, education costs are partially covered by surcharges calculated by law [21]. This surcharge not only reflects the education costs associated with medical student training, but also includes all other costs of education in a hospital, such as nursing and technical services. Additionally, simulation-based training can be costlier compared to standard teaching approaches, as seen in the case of simulation-based teaching for orthopedic residents, where the costs at the University of Toronto increased by 15 times after implementing a new curriculum [22]. In this article, we present a theoretical approximation of the costs associated with implementing a brachytherapy simulation workshop. Our cost approximation using a cost-to-benefit ratio can serve as a calculation model for similar topics.

## Materials and methods

**Fig. 1:**
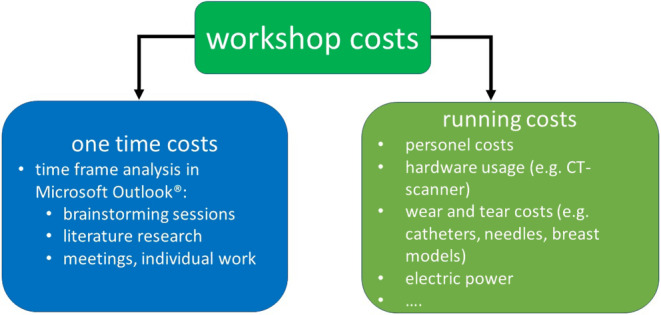
Principle of the micro-costing process, with two subcategories: costs for the workshop nascence and costs accruing with each workshop, which are further subdivided into fixed costs and variable costs

Unlike medical procedures and treatments, which have measurable financial reimbursements that allows for calculating average costs, teaching does not generate such reimbursements. In order to estimate the costs of a breast brachytherapy simulation workshop, a micro-costing approach was utilized. This approach involves estimating costs at a basic level, including supplies, software and hardware expenses, and the time invested by each member of the team. Micro-costing typically involves three stages: identifying all resources involved in the task, measuring each resource, and valuing the resources used [23]. In the field of medicine, micro-costing is a valuable tool for evaluating the costs associated with implementing new techniques, such as robotic surgery [24]. To determine the financial cost of the hands-on workshop, the analysis was divided into two sections. The first section covers the development of the workshop, including brainstorming sessions, meetings, and individual work by the team members. Meetings and other sessions involving the teaching and supporting team were recorded using Microsoft Outlook Calendar© (Microsoft Outlook 2016, Microsoft Corporation, Redmond, WA, USA), and the individual work was estimated by each participant. The second section includes the costs of conducting the workshop itself, which are incurred with each session. This breakdown is illustrated in Fig. 1.

Costs that are incurred for each additional workshop include the following: faculty hours for teaching staff (two physicians, two physicists, and one student assistant), labor costs for producing new silicon breast models, room rental and other related expenses, and wear and tear costs (such as materials that need to be replaced after a certain number of courses, e.g., silicon breast models). The costs of teaching staff were estimated using the collective agreement for German university hospitals for physicians (TV-Ärzte) and for employees (TV-L) [25, 26]. The lowest salary group for residents, specialized physicians, and medical physicists (MPE) was used for calculation purposes. Since the workshop aims to achieve specific quality goals, the participation of one physician who has completed residency in radiation oncology (RO) was considered essential. The second physician was assumed to be in the first year of residency (RE). All costs were calculated in euros (€) and can be converted to US dollars using the current conversion factor. Hourly wages were calculated based on the assumption of 2080 working hours per year [27]. To calculate the overall cost of the workshop, two separate calculations were performed. First, all costs (obtained through micro-costing) were divided by the average number of participants to determine the cost per participant. In the second calculation, the cost of improved knowledge was calculated based on the results of a knowledge progress evaluation questionnaire that used a six-point Likert scale. Improvement was determined by calculating the difference between the average post-workshop score and the pre-workshop score for each item. The costs were then divided by the improvement in scores achieved. To calculate the costs of software and hardware, factors such as initial cost, annual cost, estimated lifespan, and annual number of uses were considered. For hardware costs (e.g., CT scanner), a linear depreciation method recommended by the German Ministry of Finance was used [28].

## Results

Personnel costs were calculated as follows: the monthly cost of a radiation oncologist is € 6518.41 (based on TV-Ärzte 2022, Ä2/1), the monthly cost of a first-year resident is € 4938.79 (based on TV‑Ä Ä1/1), the monthly cost of a medical physicist is € 4188.28 (based on TV-Länder 2022, E13/1), the hourly cost of a student assistant is € 12.29 per hour according to their contract, and the cost of a precision mechanic (PM) who manufactures the in-house breast models is € 2946.46 (based on TV-Länder 2022, E8/1). The calculation for each working hour is as follows:

$$x=\frac{\textit{salary}\,per\,\textit{month}\,x12\,\textit{months}}{2080\,\text{hours}}$$

resulting in one-hour (h) costs of € 37.6 per ROh, € 28.49 per REh, € 24.96 per MPEh, and € 16.99 per PMh.

### First section—workshop nascence

First, a series of brainstorming sessions was conducted by three radiation oncologists (ROs) involved in interstitial brachytherapy. These sessions lasted for a total of three half-hour meetings, resulting in 4.5 radiation oncologist hours (ROh). Two radiation oncologists spent 2 h each, totaling 4 ROh, working on the PowerPoint® presentation, as well as the student assistant (SA) for 5 h (5 SAh). The student assistant and the precision mechanic collaborated for a total of 6 h to create the first silicone breast model. After completing the presentation and the silicone breast models, a dummy run was conducted for a total of 1.5 h, involving 3 ROh and 1.5 SAh to assess the feasibility of the scheduled 90-minute workshop duration. The student assistant also took on the responsibility of designing flyers for promotional purposes and an evaluation sheet. This activity accounted for 30 SAh (Table 1).

**Table 1:** Staff cost for brainstorming-meetings/nascence of the workshop including working on hand-outs/slides, silicone breasts, and dummy run

Staff	Radiation oncologist	Student assistant	Precision mechanic
*Hours*			
Brainstorming	0.5 h × 3 × 3 RO	–	–
PowerPoint	2 h × 2 RO	5 h	–
Dummy run	1.5 h × 2 RO	1.5h	–
Silicone models	–	6 h	6 h
Flyer design, evaluation sheet	–	30 h	–
*Total hours*	11.5 h	42.5 h	6 h
*Costs (€)*	432.4	522.32	101.94
*Total (€)*	–	–	1056.66

*RO* radiation oncologist, *h* hours

### Second section—costs per workshop

#### Hard and software

In a subsequent analysis, we endeavored to calculate the hourly costs for the primary hardware and software used in the course. This included a computed tomography (CT) scanner with associated software licenses (SyngoVia®, Siemens®, Erlangen, Germany). The initial purchase price of the CT scanner was € 400,000, and it was depreciated linearly over 8 years at a rate of 12%, resulting in an annual cost of € 48,000 [29]. Upon reaching out to the accountant responsible, it was determined that the operational lifespan of the CT scanner could be extended to 10 years, with an additional cost of € 10,000 per year for the service contract, bringing the total to € 420,000 over 10 years. In daily practice, the planning CT that was used operates in a sharing mode with the institute for radiology, so it was assumed that the CT is also in running mode for 2080 working hours a year, which is a very conservative point of view from the costs. This would result in:

$$x=\frac{\text{EUR }420.000}{2080\text{ hours }\mathrm{x}10\,\text{years}}=\text{EUR }20.19/\text{CT hour }\left(\mathrm{CTh}\right).$$

In general, electric energy costs are typically considered as overhead costs. These overhead costs can be allocated to specific cost units and translated into individual costs. One method of allocation is to use calculation rates, such as square meters, for assigning heating costs [30]. Since CTs consume a significant amount of energy, we attempted to estimate the costs of electric energy for the CT by using a 100-kWh usage over 24 h [31]. The price for 1 kWh was € 0.2565 for commercial customers and € 0.2251 per kilowatt hour for industrial customers in 2022 [32]. For our calculation, we chose a conservative rate of € 0.20 per kilowatt hour, resulting in € 20 in electric energy costs per day, equivalent to € 0.83 per hour. Therefore, 1 CT hour was valued at € 21. It should be noted that this assumption is conservative because we did not account for costs associated with water supply (machine cooling) and electric energy for lighting, among other factors. Additionally, the costs for the radiation therapy planning system (TPS) were not included in the calculation. Typically, the price of the TPS is included in the purchase or leasing contract for linear accelerators or afterloader devices, making it difficult to estimate an accurate quota (Table 2).

**Table 2:** Personnel and hardware costs accruing with each workshop in euro

*Staff*	*Radiation oncologist*	*Resident radiation oncology*	*Student assistant*	*Medical physicist*	*Total cost*
Hours	1.5 h	1.5 h	2 h	1 h	–
Cost/€	56.4	42.73	24.58	25.96	*148.67*
*Hard/software*	*CT scanner*	*TPS*	–	–	–
Hours	1 h	0.15 h	–	–	–
Cost/€	21	Withheld	–	–	*21*
*Summed total/€*	*–*	*–*	*–*	*–*	*169.67*

### Second section—costs per workshop

#### Wear and tear expenses

During each workshop session, six silicone breast models are used. Based on our experience, these need to be replaced every fifth workshop. We calculated the cost for one silicone breast model by considering the materials required for manufacturing 10 models in one production cycle. These materials include two packages of 2 kg silicone (totaling € 129.50), 1 l of silicone oil (€ 29.75), 0.5 kg of Protesil® (€ 7.50), 3 g of colors (€ 7.75), salt and gritty substances (€ 4), and silicone spray (€ 15). This sums up to a total of € 193.50 for 10 models, resulting in a wear and tear cost of approximately € 19.35 per breast model. The production of 10 breast models takes approximately 4 h of work (4 PMh), amounting to a cost of € 67.96 or approximately € 6.79 per breast model.

Therefore, costs for breast models for one workshop were

$$x=\frac{(\text{EUR }19.35\;\textit{material}+\text{EUR }6.76\;\textit{labour})\,x\,6\,\textit{models}}{5\;\textit{courses}}=\text{EUR }31.33.$$

After each production cycle of 10 models, there are unused models, since only 6 breast models are used per course. With three production cycles, a total of 18 breast models are used across three additional courses. Therefore, the three production cycles would provide breast models for 25 teaching courses. However, this would not result in significant cost reduction due to the low material requirements and the limited impact of increased purchase quantity.

**Fig. 2:**
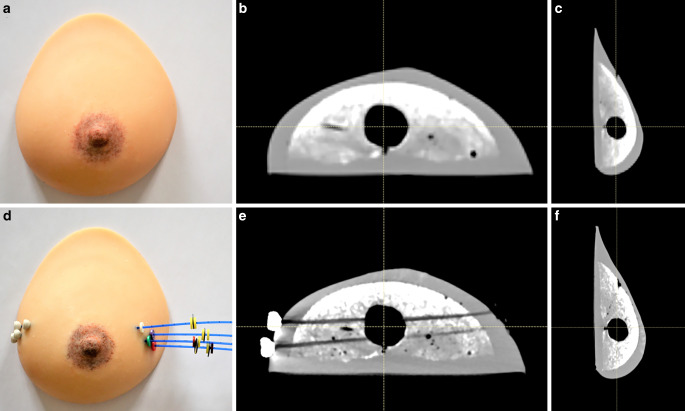
Photographic images (**a**) and CT scans of the breast model in axial (**b**) and sagittal view (**c**). The imitation of the “tumor bed” appears hypointense on CT. The catheter positions (**d**) within the breast model are visualized by CT scan (**e**–**f**)

Additional costs are incurred due to the wear and tear of brachytherapy hollow needles and single leader catheters. Three single leader catheters are implanted in each silicone breast model, and they need to be replaced after each use. Therefore, a total of 18 catheters and needles are used in each session. These catheters were supplied in a package by the manufacturer (Varian®, Palo Alto, CA), which contains five interstitial needles, five single leader catheters, and five half-moon buttons for catheter fixation. The cost of one package was assumed to be € 400 for the purpose of cost calculation, which is a conservative estimate. Based on our experience from over 10 workshops, all 20 needles and catheters (four packages) need to be used in a single workshop due to the inexperienced handling by students and residents. Although needles can theoretically be reused for teaching purposes, the combination of catheters and needles in one delivery unit renders reusability not useful. It should be noted that in our case, we received outdated sterile needles and catheters from the manufacturer specifically for the teaching course, so these costs did not apply to us. Without sponsorship, the cost of needles and catheters would have been € 1600/course at minimum, making it financially impractical to offer this workshop. Figure 2 provides a representative image of the breast models with catheters and buttons, illustrating the realistic simulation setup.

### Total costs

As per the calculations provided above, the total cost of each workshop consists of the initial setup costs as well as the recurring costs for each session. In the first year, the course was held 11 times, with some sessions aimed at promoting the course and compensating for the deficits caused by the COVID-19 pandemic. Due to positive feedback from participants, the course is planned to be held regularly six times a year for the coming years. For the purpose of calculation, the duration of the course is assumed to be 5 years. This would result in a total of 30 courses conducted, bringing the nascence costs of each workshop to € 35.22 (€ 1056.6 total nascence costs divided by 30 courses).

The remaining costs comprise the personnel and hardware costs per course, which include estimated overhead costs (Table 2, € 169.67). Additionally, there are costs for the breast models per course (€ 31.33) and the theoretical brachytherapy implantation equipment (€ 1600), as summarized in Table 3. The table presents two variants: a) with the brachytherapy equipment sponsored by the manufacturer, and b) with the equipment purchased by the faculty.

**Table 3:** Total costs per workshop in euro, scenario a) with industrial sponsoring, scenario b) without sponsoring

Total costs/course	Personnel/hardware	Breast model	Brachytherapy equipment	Nascence	Total cost
a) sponsored equipment	169.67	31.33	–	35.22	*235.55*
b) purchase equipment	169.67	31.33	1600	35.22	*1835.55*

The total cost of one hands-on teaching session is approximately € 235.55, based on conservative assumptions. This amounts to an average cost of € 39.25 per participant, considering a course with six participants. However, it is not realistic to expect that the real workshop expenses, which would include the specialized brachytherapy equipment, could have been covered by this amount.

### Cost per point Likert increment

Before and immediately after the course, participants were asked to complete a questionnaire to assess their knowledge level and evaluate the quality of the course. The results of this assessment can be found in a separate publication [19]. Participants used a six-point Likert scale consisting of nine items to self-assess their knowledge of breast brachytherapy. The sum scores of all items were calculated for statistical analysis using a non-parametric paired *t*-test. A significance level of *p* < 0.05 was considered significant. In the first year of course implementation, 70 trainees participated in 11 sessions, with 62 participants completing the questionnaires. The course participants reported significant improvements in their theoretical and practical skills as a result of the workshop. Sum scores based on Likert confidence scores were calculated for all survey statements. Prior to the workshop, the average value for the Likert confidence score was 4.7. After the workshop, the average value improved to 1.8 (*p* < 0.001), representing a 2.9-point improvement. This improvement can be seen as a benefit-to-cost ratio (bfr). The bfr, taking into account the sponsored equipment, can be calculated as follows [33, 34]:

$$bfr=\frac{\textit{value of the result}}{\textit{Cost of the investment}}\,\text{which would translate into:}$$

$$bfr=\frac{2.9\,\textit{Likert}\,\textit{points}\,}{\textit{Costs}\,per\,\textit{participant}\,\left(\text{EUR }39.25\right)}=0.073\,\text{points}/\text{EUR}.$$

We performed a modulating calculation with the theoretical assumption of reduced billing for the brachytherapy equipment of 25% of the retail price for teaching purposes. In our case, we would have needed four packages needles, catheters and buttons, € 100 each, resulting in € 400 for the brachy material plus the above mentioned € 235.55 (personnel/hardware/breast model/nascence) summing up to € 635.55 per course. This would translate into 0.027 points/€. As Likert points per cash-unit is somewhat counterintuitive, we used the reciprocal of bfr, which can simply be seen as a cost-to-benefit ratio, CBFR:

$$CBFR=\frac{\textit{Costs}\,per\,\textit{participant}\,\left(\text{EUR }39.25\right)}{2.9\,\textit{Likert}\,\textit{points}\,}$$

CBFR would be € 13.53 for each Likert point increment.

One way to evaluate the effectiveness and cost-effectiveness of a workshop or training program is through use of the transfer effectiveness ratio (TER), which was initially developed for assessing aircraft pilot training [35]. The TER measures the extent to which training translates into improved performance in real-world tasks. It is calculated as follows:

$$TER=\frac{Tc-Tx}{X}$$

Where Tc represents the time or number of trials needed for a control or baseline group to achieve criterion performance (in this case, the conventional teaching time in the operating room); Tx represents the time or number of trials needed for an experimental group (in this case, the participants) to achieve criterion performance after x amount of time or number trials using simulation or another instructional approach of interest; X represents the time or number of trials spent by the experimental group using simulation (in our case, 30 min of catheter implantation time per participant). The TER indicates how many trials or units of time are saved for every unit of simulation-based training, in order to reach the desired objective experience [36]. In our case, the TER indicates how many trials (or how much time) are saved in achieving breast brachytherapy performance for every unit of workshop training invested. Calculation of the TER was based on the assumption and experience that conventional training needs to be conducted during a real brachytherapy catheter implantation in the operating room. Tc was considered as the learning time required for the first safe catheter implantation and estimated to be 30 min. Tx represents the time for the same routine handling after the initial workshop training with residents, and was estimated to be 10 min. By teaching six workshop participants at a time and providing them with an understanding of the implantation procedure, the initial 30-min teaching time could be reduced to 10 min:

$$TER=\frac{Tc(30\,min)-Tx(10\,min)}{30\,min}$$

The TER is 0.66, meaning that for every 10 h of simulation-based teaching, there is a saving of 6.6 h. The TER is calculated based on the cost savings achieved by using simulation training instead of expensive real-time training in the operating room. The specific TER threshold depends on the subject being investigated [37]. To approximate costs associated with training in the operating room and thereby to calculate the saved 6.6 h per 10 h of training is far beyond the scope of this article. Therefore, the advantage of our simulation-based approach should be viewed in terms of the widespread dissemination of brachytherapy knowledge per time, the motivation of the participants, and the possibility to give the participants some self-confidence for their first breast brachytherapy under supervision.

## Discussion

The results of the course evaluation demonstrate that this hands-on workshop, which requires significant personal investment of time, clearly enhances participants’ knowledge and skills in the taught field. Courses like ours can partially address the issue of insufficient didactic and procedural training [12]. Our course was intended to be held once per participant. However, one could discuss whether the participants manual skills would improve if the implantation procedure were to be trained several times, but this is beyond the scope of the current work. In Germany, current reform efforts in medical education, such as the *Masterplan Medizinstudium 2020* and the revision of the National Competence-Based Catalogue of Learning Objectives in Medicine (NKLM), emphasize the importance of consistent practice and competence-oriented teaching. Simulation-based training, rooted in active learning theories from cognitive and learning sciences, is increasingly sought after in modern medical education across various specialties [38]. In the field of radiation oncology, there is a significant demand for more practical lessons in training healthcare professionals [39]. However, despite their didactic advantages, the adoption of simulation-based teaching concepts in radiation oncology has been limited, possibly due to the associated requirements for personnel, time, and financial resources. The costs associated with simulation-based training in particular pose a major obstacle [40]. Significant financial investments are necessary to establish the required infrastructure, support staff, faculty time, and operating materials [41]. A study conducted in the United States found that academic medical centers were 44% more expensive and other teaching hospitals were 14% more costly compared to non-teaching hospitals. The authors attributed the majority of this cost difference to the intensity of teaching [42].

In a micro-costing approach for estimating hospital costs for appendectomy in a cross-European context, Schreyögg excluded teaching hospitals to avoid the bias of more costly cases due to higher resource intensity in these hospitals [43]. Zendejas et al. conducted a systematic review and found that only 1.6% of studies provided any cost comparison when examining simulation-based training methods compared to other instructional methods in medical education [41]. In this work, we tried to address the theoretical costs of such a workshop. By a micro-costing approach, we were able to estimate the costs of our hands-on workshop at about € 235 per course and € 39 per participant with industrially sponsored material supply and showed that these costs increase to levels not fundable when retail prices are used for calculation. The workshop was systematically evaluated by the participants, and we were able to show a significant improvement in skills. We were able to calculate a cost-to-benefit ratio of € 13.53 for each Likert point improvement.

Of course, there are some limitations to these calculations. Firstly, it should be noted that all the brachytherapy catheters, needles, and buttons used in the course were sponsored as sterile off-dated materials from the manufacturer, provided to us for free. Secondly, the costs of software such as the radiation therapy planning system (TPS) were not considered. This decision was made due to the difficulty in obtaining realistic quotes for this software, as it is typically bundled with the afterloader that was not used in the course. As it was possible to obtain an agreement for industrial sponsoring of the needles and catheters, if necessary, one could try to get a TPS sponsored that is only licensed for teaching purposes. Additionally, the costs of the lecture hall were not considered, as it is provided free of charge for educational and teaching purposes in accordance with house policy. We did try to estimate the cost of using the CT scanner, as the need for servicing depends on the accumulated working hours. However, a conservative cost approach was taken, assuming a high volume of 2080 working hours, and common costs, e.g., water supply, were withheld. The cost of one CT working hour was calculated at € 21/hour, and this component accounted for only 10% of the total costs per course. It is evident that the main cost drivers are the human work involved and the costs of the brachytherapy equipment, which is consistent with the findings of published literature [41]. It should be noted that for calculation purposes, the lowest salary group for residents, consultants, and medical physicists was used for the calculation. As such courses are usually not delivered by the least experienced staff, this may lead to an underestimation of the real costs. Every institution that wants to calculate their respective costs has to use the paid salaries in the formulas. Without the sponsorship of the brachytherapy materials, the implementation of this course would not have been possible. It should be noted that at our center, breast brachytherapy is performed on a routine basis, which leads to a relatively high demand for brachytherapy equipment and thereby intensive contact to the industry and also some kind of customer power. Besides getting material for free, institutions could try to get non-sterile catheters for a reduced prize, which of course has to be considered when performing micro-costing. Participants of our workshop did not have to pay any fees. As an option, participants could be charged some amount to cover the costs of material, but one should be aware of the fact that tax regulations for income have to be considered. However, it is clear that the real costs of such a high-end teaching course cannot be accurately reflected in the calculation of surcharges for teaching hospitals.

However, our cost calculation appears to be a realistic approximation, considering the University of Toronto’s report of a significant increase in costs for teaching orthopedic residents, from $ 11,140 to $ 158,050, after implementing a competency-based curriculum [22]. Teaching is undeniably a crucial part of the mission of teaching hospitals. However, intensive teaching courses, such as the one described here or others mentioned in the literature, cannot be conducted concurrently with patient care, which is the primary source of income for hospitals. In essence, hospitals can be viewed as companies that provide goods and services for patient treatment. Furthermore, our workshop involved not only physician resources but also other professionals like medical physicist experts and precision mechanics. In Germany, some initiatives have been taken by policymakers to address the challenge of intensive modern teaching, such as providing support through funding initiatives like the fellowship for innovations in digital teaching supported by the Free State of Thuringia. Additionally, universities themselves or non-governmental organizations may offer financial support for innovative teaching concepts [44]. Fundraising in education is an intriguing concept that has the potential to be beneficial in offsetting the rising costs associated with modern educational approaches [44]. In this regard, medical educators can play a pivotal role as connecting networkers and knowledge brokers. They have the ability to provide valuable insights into the faculty’s strategic priorities and serve as stewardship officers, facilitating interactions between donors, alumni, and the institution. These interactions allow for updates on the institution’s latest accomplishments and offer opportunities to address any queries from donors and alumni [45]. However, the issue of high material costs persists for many hands-on workshops, as they require specialized equipment to provide a realistic learning experience [45]. Since the COVID-19 pandemic, online teaching has gained significance and has shown high participant satisfaction. Nonetheless, it should be noted that online formats are not suitable for developing practical skills [46]. Given the overall societal importance of teaching qualified medical professionals, the question to be asked is not “to teach or not to teach?” [18], but rather how we can collaborate and cooperate to effectively teach within our available resources.

## Conclusion

Teaching participants through a hands-on workshop in multicatheter breast brachytherapy proved to be highly effective in enhancing practical skills. To estimate the costs associated with this innovative teaching format, a micro-costing approach was utilized. To support the implementation of such time-consuming and financially demanding teaching formats, internal university or faculty fundraising can be beneficial. Additionally, funding from third parties or industrial partners may be available. The micro-calculation example, including the calculation of a cost–benefit ratio (CBFR), can be applied to other formats to estimate costs more effectively.

### Supplementary Information

Questionnaire for the participants to self-assess knowledge level
