# Supplementary material for: Cost–benefit ratio of modern medical education using micro-costing: a model calculation using the example of an innovative breast brachytherapy workshop
Source: Strahlenther Onkol. 2024 Feb 28;200(4):325–34. doi: 10.1007/s00066-024-02218-6 (PMC10965664; doi:10.1007/s00066-024-02218-6)
Supplement: Supplementary file 1 — Questionnaire for the participants to self-assess knowledge level [file 66_2024_2218_MOESM1_ESM.docx]

Evaluation: Workshop – Brachytherapie

# Please mark before the workshop !

| Even **before** the workshop… | **1 – I completely**  **agree** | **2 – I mostly agree** | **3 – I vote rather too** | **4 I vote rather not** | **5 I mostly do not agree** | **6 – Do not agree at all** |
| --- | --- | --- | --- | --- | --- | --- |
| I was able to explain the basics of the etiology and diagnosis of early-stage breast cancer | **☐** | **☐** | **☐** | **☐** | **☐** | **☐** |
| I was able to describe the guideline-based treatment of early-stage breast cancer | **☐** | **☐** | **☐** | **☐** | **☐** | **☐** |
| I was familiar with various areas of application of brachytherapy | **☐** | **☐** | **☐** | **☐** | **☐** | **☐** |
| I was able to characterize the patient population eligible for partial breast irradiation | **☐** | **☐** | **☐** | **☐** | **☐** | **☐** |
| I was able to describe the steps of brachytherapy | **☐** | **☐** | **☐** | **☐** | **☐** | **☐** |
| I can compare brachytherapy with percutaneous radiation in terms of advantages and disadvantages | **☐** | **☐** | **☐** | **☐** | **☐** | **☐** |
| I was able to implant single leader catheters into a breast model | **☐** | **☐** | **☐** | **☐** | **☐** | **☐** |
| I was able to explain the importance of adequate catheter implantation for radiation planning | **☐** | **☐** | **☐** | **☐** | **☐** | **☐** |
| I was able to describe the process of radiation planning and delivery | **☐** | **☐** | **☐** | **☐** | **☐** | **☐** |
|  |  |  |  |  |  |  |

# Please mark After the workshop!

| Now, **after** the workshop… | **1 – I completely**  **agree** | **2 – I mostly agree** | **3 – I vote rather too** | **4 I vote rather not** | **5 I mostly do not agree** | **6 – Do not agree at all** |
| --- | --- | --- | --- | --- | --- | --- |
| I can explain the basics of the etiology and diagnosis of early-stage breast cancer | **☐** | **☐** | **☐** | **☐** | **☐** | **☐** |
| I can describe the guideline-based treatment of early-stage breast cancer | **☐** | **☐** | **☐** | **☐** | **☐** | **☐** |
| I am familiar with various areas of application of brachytherapy | **☐** | **☐** | **☐** | **☐** | **☐** | **☐** |
| I was able to characterize the patient population eligible for partial breast irradiation | **☐** | **☐** | **☐** | **☐** | **☐** | **☐** |
| I can describe the steps of brachytherapy | **☐** | **☐** | **☐** | **☐** | **☐** | **☐** |
| I can compare brachytherapy with percutaneous radiation in terms of advantages and disadvantages | **☐** | **☐** | **☐** | **☐** | **☐** | **☐** |
| I am able to implant single leader catheters into a breast model | **☐** | **☐** | **☐** | **☐** | **☐** | **☐** |
| I can explain the importance of adequate catheter implantation for radiation planning | **☐** | **☐** | **☐** | **☐** | **☐** | **☐** |
| I am able to describe the process of radiation planning and delivery | **☐** | **☐** | **☐** | **☐** | **☐** | **☐** |
|  |  |  |  |  |  |  |

# Please mark After the workshop!

|  | **1 – I completely**  **agree** | **2 – I mostly agree** | **3 – I vote rather too** | **4 I vote rather not** | **5 I mostly do not agree** | **6 – Do not agree at all** |
| --- | --- | --- | --- | --- | --- | --- |
| The learning objectives were clearly defined. | **☐** | **☐** | **☐** | **☐** | **☐** | **☐** |
| The learning content was adequately illustrated | **☐** | **☐** | **☐** | **☐** | **☐** | **☐** |
| The lecturers ensured an appreciative learning atmosphere | **☐** | **☐** | **☐** | **☐** | **☐** | **☐** |
| The lecturers gave specific work assignments | **☐** | **☐** | **☐** | **☐** | **☐** | **☐** |
| Catheter implantation on the breast model was suitable for achieving the learning objectives | **☐** | **☐** | **☐** | **☐** | **☐** | **☐** |
| By visting the CT and brachytherapy department, I was able to deepen my learning objectives | **☐** | **☐** | **☐** | **☐** | **☐** | **☐** |
| The workshop was of high didactic quality | **☐** | **☐** | **☐** | **☐** | **☐** | **☐** |
| Course time management was appropriate | **☐** | **☐** | **☐** | **☐** | **☐** | **☐** |
| The course fostered my interest in the topic | **☐** | **☐** | **☐** | **☐** | **☐** | **☐** |
| I highly value the knowledge I gained from the course. | **☐** | **☐** | **☐** | **☐** | **☐** | **☐** |
| My overall impression: | **☐** | **☐** | **☐** | **☐** | **☐** | **☐** |

# WHAT DID YOU LIKE Most?

# WHERE DO YOU SEE POTENTIAL FOR IMPROVEMENT?
